# Disruption of the Auxin Gradient in the Abscission Zone Area Evokes Asymmetrical Changes Leading to Flower Separation in Yellow Lupine

**DOI:** 10.3390/ijms21113815

**Published:** 2020-05-27

**Authors:** Agata Kućko, Emilia Wilmowicz, Wojciech Pokora, Juan De Dios Alché

**Affiliations:** 1 Department of Plant Physiology, Institute of Biology, Faculty of Agriculture and Biology, Warsaw University of Life Sciences-SGGW, Nowoursynowska 159, 02-776 Warsaw, Poland; kuckoa@poczta.onet.pl; 2 Chair of Plant Physiology and Biotechnology, Faculty of Biological and Veterinary Sciences, Nicolaus Copernicus University, 1 Lwowska Street, 87-100 Toruń, Poland; 3 Department of Plant Physiology and Biotechnology, Faculty of Biology, University of Gdańsk, Wita Stwosza 59, 80-308 Gdańsk, Poland; wojciech.pokora@biol.ug.edu.pl; 4 Plant Reproductive Biology and Advanced Microscopy Laboratory, Department of Biochemistry, Cell and Molecular Biology of Plants, Estación Experimental del Zaidín, Spanish National Research Council (CSIC), Profesor Albareda 1, E-18008 Granada, Spain; juandedios.alche@eez.csic.es

**Keywords:** abscisic acid, abscission zone, auxin gradient, ethylene, organ separation, reactive oxygen species, yellow lupine, yielding

## Abstract

How auxin transport regulates organ abscission is a long-standing and intriguing question. Polar auxin transport across the abscission zone (AZ) plays a more important role in the regulation of abscission than a local concentration of this hormone. We recently reported the existence of a spatiotemporal sequential pattern of the indole-3-acetic acid (IAA) localization in the area of the yellow lupine AZ, which is a place of flower detachment. In this study, we performed analyses of AZ following treatment with an inhibitor of polar auxin transport (2,3,5-triiodobenzoic acid (TIBA)). Once we applied TIBA directly onto the AZ, we observed a strong response as demonstrated by enhanced flower abscission. To elucidate the molecular events caused by the inhibition of auxin movement, we divided the AZ into the distal and proximal part. TIBA triggered the formation of the IAA gradient between these two parts. The AZ-marker genes, which encode the downstream molecular components of the inflorescence deficient in abscission (IDA)-signaling system executing the abscission, were expressed in the distal part. The accumulation of IAA in the proximal area accelerated the biosynthesis of abscisic acid and ethylene (stimulators of flower separation), which was also reflected at the transcriptional level. Accumulated IAA up-regulated reactive oxygen species (ROS) detoxification mechanisms. Collectively, we provide new information regarding auxin-regulated processes operating in specific areas of the AZ.

## 1. Introduction

Abscission is a developmentally programmed phenomenon that facilitates the shedding of damaged, senescent, or unnecessary organs, such as leaves, petals, or in some cases, infected parts of a plant. Under certain circumstances, e.g., unfavorable environmental or endogenous conditions, such as drought or nutritional limitations, even necessary organs are shed. Importantly, in a similar way, whole flowers or young fruit detachment may occur. A serious consequence of this is reduced yielding in many crop species (reviewed by References [1,2]). Regardless of the factor that induces abscission, this process always takes place in a specialized group of cells that form what is called an abscission zone (AZ) [3]. For a few reasons, the most recent studies about organ abscission are mainly focused on the events that accompanied the activation of separation processes directly taking place in the AZ cells. First, abscission-related transformations are limited to the AZ and are highly-specific to this structure, thus they are not observed in the tissues located above and below. Second, AZ becomes competent at responding to abscission signals. It is extremely sensitive to exogenous stimulators of abscission, such as plant hormones and their inhibitors. Finally, most of the abscission-induced genes are expressed directly in the AZ following the activation of the separation. The precisely regulated timing of organ detachment is determined by the metabolic activity of the AZ cells, which highly depends on the presence of phytohormones, including abscisic acid (ABA), ethylene (ET), and auxin [4,5]. 

In recent years, our knowledge about separation processes has been improved mainly due to advances in genetic, molecular, and biochemical methods. Next to hormones, many additional molecules have been identified as playing a crucial role in abscission-accompanying events. Among them, a small signaling peptide (inflorescence deficient in abscission (IDA)) and its receptor HAESA/HAESA-like function in a genetic pathway that also involves MAP kinases, which belong to the most important plant-signaling networks involved in response to changing environmental conditions. MPK-mediated protein phosphorylation determines their activity, stability, and localization, which in turn modulates gene expression and triggers phytohormone pathways [6]. It has been reported that the formation of the IDA-HAE/HSL2 complex led to the release of KNAT1 (knotted-like from *Arabidopsis thaliana* 1) transcription factor, which is a repressor of *KNAT2* and *KNAT6* expression. KNAT2/KNAT6 mediates the activation of genes involved in the dissolution of pectin-rich middle lamellae, and consequently in the disintegration of AZ tissues [7,8,9].

Reactive oxygen species (ROS), e.g., superoxide anion (O^2−^), hydrogen peroxide (H_2_O_2_), hydroxyl radical (OH^−^), and singlet oxygen (^1^O_2_), are important signaling messengers produced in the plant cell wall to modify its components via processes of stiffening/softening. It has been established that exogenous ROS can induce organ abscission, while the application of antioxidants and ROS scavengers inhibits this process [10,11,12,13,14]. Every cell possesses several mechanisms, both non-enzymatic and enzymatic, to regulate ROS levels. An active antioxidant system includes mainly superoxide dismutases (SODs), catalases (CATs), and peroxidases (POXs) (reviewed by Kärkönen and Kuchitsu [15]). SODs function as the first line of defense against ROS. These enzymes have been classified into three groups according to their metal cofactor: copper-zinc (Cu/Zn-SOD), manganese (Mn-SOD), and iron (Fe-SOD) [16]. 

Yellow lupine (*Lupinus luteus* L.) is an excellent model for studying abscission-related changes. It is an agronomically important species that is cultivated for food and feed in many regions of the globe [17,18]. Thus, scientific data on this issue could provide significant information not only for researchers but also for breeders to improve yielding potential. Special attention should be given to the fact that the most limiting factor for yellow lupine productivity is excessive flower abscission. In turn, this makes lupine a useful model to elucidate the molecular basis of this phenomenon. In this species, the distal side of the flower AZ is connected to the pedicel, while the proximal is adjacent to the stem [19,20,21]. Our comprehensive studies revealed that lupine AZ is highly sensitive to ABA and ET [22,23]. Intriguingly, the AZ is a tiny structure; however, the changes taking place in its distal and proximal parts can also be different and specific [20,24]. We previously showed that auxin distribution varied in the distal and proximal area of AZ following the early and late steps of abscission [20]. A direct consequence of the changing balance of IAA (indole-3-acetic acid) is the induction of the elements of the ET biosynthesis pathway [20]. This gaseous phytohormone acts as an executor of abscission and causes flower detachment [23]. Generally, ET regulates organ abscission via induction of the expression of genes encoding cell-wall-modifying enzymes, as well as through increasing their activity, and consequently degradation of the middle lamella [25]. The results of research carried out on other plant species indicate that the depletion of the auxin level in the AZ renders the cells more sensitive to ET (reviewed by References [26,27]). Moreover, we have shown that the exposure of lupine AZ to ET significantly affected IAA localization in the whole AZ in a time-dependent manner [20]. 

We have already indicated that the flower separation process in lupine is associated with a substantial accumulation of *blade-on-petiole* (*LlBOP*), *inflorescence deficient in abscission-like* (*LlIDL*), *receptor-like protein kinase HSL* (*LlHSL*), and *mitogen-activated protein kinase 6* (*LlMPK6*) transcripts [20,21,28,29]. All these genes encode succeeding elements of the AZ activation molecular pathway. Furthermore, *LlBOP* and *LlIDL* expressions are regulated by ET and ABA which are strong stimulators of flower detachment [28,29]. Abscission is also linked to the up-regulation of *zeaxanthin epoxidase* (*LlZEP*) gene in ABA biosynthesis and *aminocyclopropane-1-carboxylic acid synthase* (*LlACS*) and *aminocyclopropane-1-carboxylic acid oxidase* (*LlACO*) genes in ET biosynthesis [22,23]. The induction of the molecular pathway that stimulates transformations in the AZ leading to abscission is accompanied by specific cellular features that also provide information about metabolic changes. We showed that cells of the AZ isolated from naturally abscised flowers divided intensively and were linked by numerous plasmodesmata, which suggest the possibility of intercellular communication [29]. The cytoplasm of active AZ cells was dense, heterogeneous, and enriched in proteins, suggesting the de novo synthesis of enzymes crucial for organ separation [30]. Analyses performed using transmission electron microscopy also revealed that these cells were progressively degraded. The observation of cell organelles, such as chloroplasts and nuclei, indicated DNA fragmentation and the possibility of engaging the mechanisms of programmed cell death (PCD), which are probably caused by the formation of intensively synthesized ROS [19,29]. Following stress-induced AZ activation, the content of hydrogen peroxide (H_2_O_2_), the CAT activity, and the localization significantly changed, which indicated modifications in the redox balance [21]. Furthermore, we observed many vesicles in the cytoplasm of active AZ cells that can mediate the transport of necessary substances, including hydrolytic enzymes. This can suggest a high metabolic activity of the AZ cells and a strong connection between them. In turn, the inactive AZ structure was completely different. These cells were rounded, loosely arranged, filled with large vacuoles, and contained few vesicles in the thin layer of cytoplasm. Importantly, we observed no cell divisions in such cells [29]. 

Summarizing, a multilevel and complex mechanism of phytohormonal networking is active in the regulation of organ abscission events. However, the mechanisms governing specific differences between distal and proximal AZ areas are not yet clear. Although the involvement of auxin in abscission-related events has been analyzed for the last few decades, still little is known about the molecular, biochemical, and cellular changes caused by the disturbance of polar auxin transport (PAT), which is a factor responsible for the organ maintenance of the plant. For this study, we decided to continue our previous considerations related to auxin balance in the AZ area and answer another scientific question. Using an inhibitor of PAT, namely 2,3,5-triiodobenzoic acid (TIBA), we analyzed the molecular events related to flower abscission that were evoked by the disruption of auxin movement through the AZ. Importantly, we described changes related to TIBA-induced abscission in the different areas of the flower AZ. The present paper provides completely novel information about tissue-specific changes within the AZ, which is a tiny but extremely significant fragment of a plant.

## 2. Results

### 2.1. Inhibition of Auxin Transport through the AZ Cells Determined the Flower Shedding

**Figure 1:**
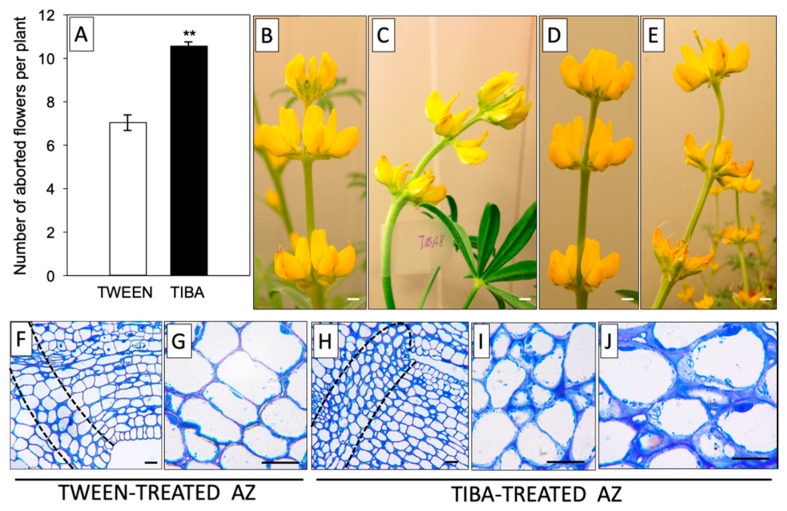
The inhibitor of auxin transport induced flower abscission in yellow lupine. For the analyses, a solution of TIBA (2,3,5-triiodobenzoic acid, 0.05 mM in 0.05% Tween 20) or 0.05% Tween 20 was applied directly to the inactive flower abscission zone (AZ). The flower abscission rate was monitored for 120 h after the TIBA or Tween 20 application (**A**). Distortion of the stem evoked by inhibition of indole-3-acetic acid (IAA) polar transport; pictures were taken 8 h (**C**) or 24 h (**E**) after TIBA treatment and 8 h (**B**) or 24 h (**D**) after Tween 20 application. Histological analysis was made to compare the AZ cells treated with Tween 20 solution (**F**–**G**) and AZ cells activated using TIBA (**H*–*J**). Observations were performed 24 h after the application of substances. The dotted lines delimit the AZ regions. At least 15 plants were used in the physiological assay. Values are presented as the mean ± SE. The experiment was performed in three independent biological replications. Scale bars: 10 mm (**B**–**E**) and 40 µM (**F**–**J**).

Based on previous reports, it is known that it is not the total IAA content, but rather the maintenance of a proper ratio of this phytohormone between different areas of the AZ that is the most relevant factor determining the time of abscission [31,32]. Our earlier immunocytochemical analyses provided evidence for the existence of a spatiotemporal sequential pattern of the IAA gradient related to the flower abscission process in *L. luteus* [20]. In this work, we continued this subject and tested the influence of a PAT inhibitor on the flower abortion rate. As shown in Figure 1A, TIBA treatment significantly accelerated the abscission of flowers. The effect of the PAT inhibitor, as shown by the presence of curved stems, was already visible 8 h after application and was maintained in the subsequent hours (up to 24 h) (Figure 1C,E). By contrast, the control inflorescences were straight and showed no changes (Figure 1B,D). With the use of the described methodology for direct TIBA application on the AZ, we induced specific changes at the cellular level, such as the presence of less densely packed cells that contained a higher number of granules and vesicles (Figure 1H–J) compared to the control with an inactive, TWEEN-treated AZ (Figure 1F,G). In brief, these results suggest that delayed IAA transport through the AZ cells evoked by TIBA was a sufficient factor for inducing the symptoms preceding the abscission of flowers.

### 2.2. Disruption of Polar Auxin Transport Significantly Influenced the IAA Content in Different Areas of the Flower AZ

**Figure 2:**
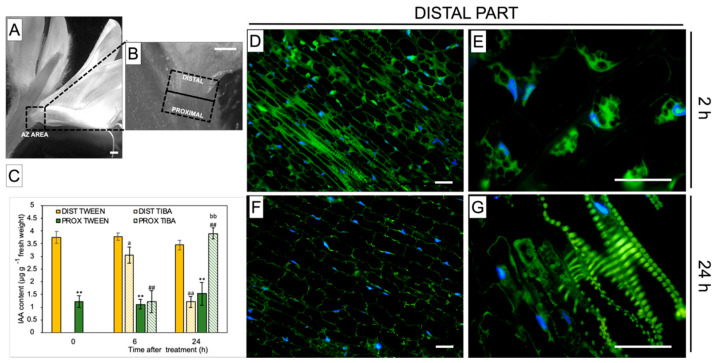
Different AZ regions exhibiting the specific gradient of IAA, which was reversed by an exogenous polar auxin transport inhibitor An image of yellow lupine flower presenting the abscission zone (AZ) area, which is marked by a black dashed-line box (**A**). Magnification indicating various regions of the AZ used for analyses (**B**). Distal and proximal parts are located ≈1 mm above and ≈1 mm below the AZ, respectively (marked by dotted black lines). The levels of IAA in the different regions of the AZ following TIBA (2,3,5-triiodobenzoic acid) application (**C**). A solution of TIBA (0.05 mM in 0.05% Tween 20) or 0.05% Tween 20 was applied locally on the inactive AZ. The tissue fragments containing AZ were dissected 6 h or 24 h after treatments and divided into a proximal (PROX) and a distal (DIST) part. Averages and SD of three biological replicates. *t*-test: ** *p* < 0.01, proximal versus distal part in the Tween 20-treated AZ for different times after application; ^##^ *p* < 0.01, proximal versus distal part in the TIBA-treated AZ for different times after application; ^a^ *p* < 0.05 and ^aa^ *p* < 0.01, TIBA-treated distal part versus Tween 20-treated distal part for different times after application; ^bb^ *p* < 0.01, TIBA-treated proximal part versus Tween 20-treated proximal part for different times after application. The localization of IAA in the distal area of the AZ 2 h and 24 h after the TIBA local application (**D**–**G**). Images (**E**) and (**G**) present higher magnification images of (**D**) and (**F**), respectively. The green fluorescence corresponds to the IAA presence. Nuclei were visualized using a blue-fluorescent DAPI stain. Scale bars: 2 mm (**A**,**B**) and 40 µM (**D**–**G**).

For further examination of the TIBA action, we divided the whole AZ area into two parts: distal (upper part, closer to the flower) and proximal (lower part, above the AZ, and closer to the stem), as illustrated in Figure 2A,B. By measuring the concentration of IAA distributed among these two parts in control plants, we found that the hormone was substantially accumulated in the distal part of inactive, non-treated AZ cells (0 h) (Figure 2C). Once TIBA was applied, the content of IAA slightly decreased in the distal region (2 h) and dropped rapidly after 24 h. At the same time (24 h), we observed a significant increase of phytohormone content in the proximal AZ area, which was even comparable to that noticed for the distal part of the inactive AZ. Using a methodology of IAA immunolocalization, our subsequent investigation strongly supported the fact that TIBA application led to a gradual decrease in the phytohormone content in the distal region of AZ during the 24 h after treatment (Figure 2F) when compared to the first 2 h (Figure 2D). Initially, IAA was observed in the cytosol in the different poles of the cells (Figure 2E), but later, the phytohormone was detected only in cells located close to the vascular bundles (Figure 2G). Taken together, PAT was effectively disrupted throughout the inactive AZ via the local application of TIBA, which reversed the spatial gradient of IAA maintained between distal and proximal regions of AZ. This, in consequence, led to flower abscission (Figure 1A). 

### 2.3. Inhibition of Auxin Transport through the AZ Cells Affected the Expression of Molecular Components of the Pathway Responsible for AZ Function

**Figure 3:**
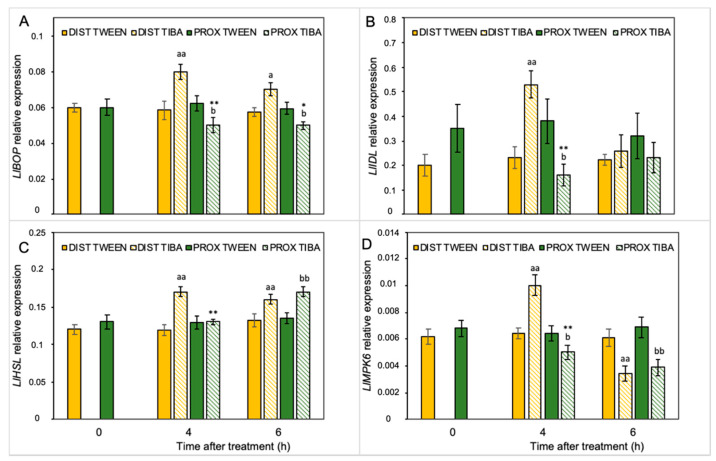
Different parts of the AZ treated with polar auxin transport inhibitor were characterized by a specific pattern of expression of abscission-related genes. The relative transcript levels of *LlBOP* (*blade-on-petiole*) (**A**), *LlIDL* (*inflorescence deficient in abscission*) (**B**), *LlHSL* (*HAESA-like*) (**C**), and *LlMPK6* (*mitogen-activated protein kinase 6*) (**D**) were investigated. A solution of 2,3,5-triiodobenzoic acid (TIBA, 0.05 mM in 0.05% Tween 20) or 0.05% Tween 20 solution was applied directly on the inactive flower abscission zone (AZ). Then, the tissue sections containing the AZ (≈1 mm below and ≈1 mm above) were dissected 4 h or 6 h after treatments and divided into a proximal (PROX) and a distal (DIST) part for further RNA isolation. Gene expression was examined using qPCR analysis. *LlACT* (*actin*) was used as an internal control for the normalization of gene expression. Values are presented as the mean of three biological replicates. Error bars indicate SEs. *t*-test: ** *p* < 0.01 and * *p* < 0.05, proximal versus distal part in the Tween 20-treated AZ for different times after application; ^aa^ *p* < 0.01 and ^a^ *p* < 0.05, TIBA-treated distal part versus Tween 20-treated distal part for different times after application; ^bb^ *p* < 0.01 and ^b^ *p* < 0.05, TIBA-treated proximal part versus Tween 20-treated proximal part for different times after application.

Our previous analyses performed on yellow lupine resulted in the identification of several abscission-associated genes that were directly up-regulated in AZ cells during their activation. Among them, *LlBOP*, *LlIDL*, *LlHSL*, and *LlMPK6* are those of major importance [19,21,28,29]. Taking into consideration the fact that IAA is the molecule involved in separation processes, and that TIBA action significantly affects the AZ-specific distribution of auxin, here we aimed to determine the influence of PAT disruption on the transcriptional activity of these molecular elements in the different areas of the AZ. We analyzed the expression pattern just 4 h and 6 h after TIBA application because protein products of these genes are involved in the initial steps of flower separation. Among them, *LlBOP* showed an opposite pattern of expression in both regions in the presence of TIBA (Figure 3A): on the distal side, the mRNA content increased, while it decreased in the proximal part. Interestingly, the relative transcript level of *LlBOP* remained unchanged in both parts of the non-treated AZ (0 h) (Figure 3A). The same relationships were noticed in the case of *LlIDL* mRNA (Figure 3B). When the TIBA was applied, we observed that the gene expression had already drastically dropped already in the fourth hour and maintained its low level for the rest of the examined timeframe. In the proximal region, the *LlIDL* expression was significantly higher when the sample was taken 4 h after the TIBA treatment (Figure 3B). It seems that the transcriptional activity of this gene was only transiently up-regulated (4 h). When we analyzed the abundance of *LlHSL* transcripts, we found that this gene was up-regulated in both the distal part (4 and 6 h) and the proximal region (6 h) in response to the TIBA treatment (Figure 3C). There were no significant differences between the two parts of the non-treated AZ (0 h) in the case of *LlHSL* (Figure 3C) and *LlMPK6* (Figure 3D). Special sensitivity toward the inhibition of PAT was noticed for the *LlMPK6* gene. Initially, its expression rose in the distal side (4 h), and then it dropped (6 h). In the proximal part, it was negatively regulated over time (Figure 3D). The results obtained strongly support the fact that both parts of the flower AZ were characterized by triggering a differential and specific pattern of expression of the subsequent components of the genetic pathway that is responsible for AZ activation after the inhibition of polar auxin transport.

### 2.4. Inhibition of Polar Auxin Transport in AZ Cells Caused Area-Specific Induction of ABA and ET Biosynthesis Pathways

**Figure 4:**
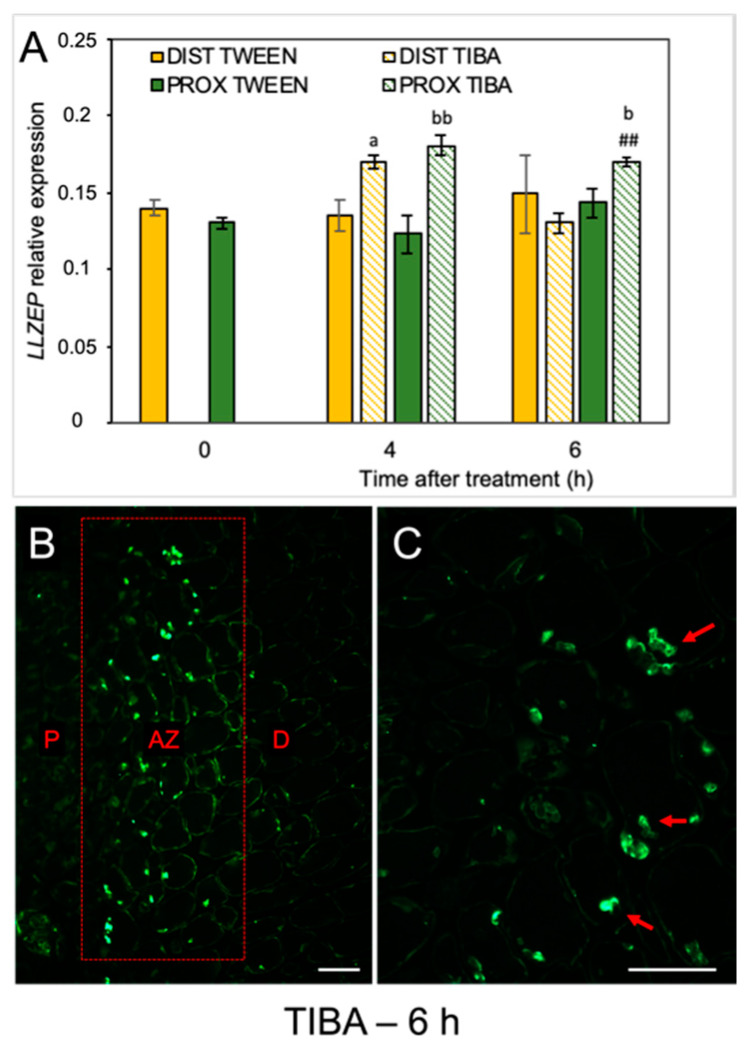
The disruption of polar auxin transport induced the *LlZEP* gene and altered the ABA localization in the abscission zone area. The relative level of *LlZEP* (*zeaxanthin epoxidase*) transcripts was investigated using qPCR (**A**). A solution of 2,3,5-triiodobenzoic acid (TIBA, 0.05 mM in 0.05% Tween 20) or 0.05% Tween 20 was applied directly on the inactive flower abscission zone (AZ). Subsequently, the tissue fragments containing the AZ (≈1 mm below and ≈1 mm above) were dissected 4 h or 6 h after treatments and divided into a proximal (PROX) and a distal (DIST) part for further RNA isolation. *LlACT* (*actin*) was used as an internal control for the normalization of gene expression. Values represent mean ± SD (n = 3). Error bars indicate SEs. *t*-test: ^a^ *p* < 0.05, TIBA-treated distal part versus Tween 20-treated distal part for different times after application; ^bb^ *p* < 0.01 and ^b^ *p* < 0.05, TIBA-treated proximal part versus Tween 20-treated proximal part for different times after application, ^##^ *p* < 0.01, proximal versus distal part in the TIBA-treated AZ for different times after application. Immunofluorescence localization of ABA in the AZ area (**B**). Magnified region of the AZ (**C**). P—proximal side, D—distal side. Observations were carried out 6 h after the TIBA application. The presence of green fluorescence indicates ABA localization (marked by red arrowheads). Scale bars: 40 µM.

When the molecular pathway responsible for AZ activation is induced, changes in the phytohormonal balance in AZ cells concomitantly occur. Plant hormones can transmit signals and/or could be the final effectors of abscission, such as ET, which activate the hydrolytic enzymes during organ separation [33,34,35]. As we previously mentioned, the flower AZ in lupine is highly sensitive to the exogenous ABA and ET [22,23]. We revealed that ABA is accumulated in the AZ isolated from naturally abscised flowers. The phytohormone was abundant, especially in the cytoplasm of the divided cells of the active AZ [22]. Furthermore, when we induced flower separation using drought stress, the *LlZEP* involved in the ABA biosynthesis was up-regulated in the AZ [21]. This is why the next set of analyses addressed the possibility that the inhibition of PAT could alter ABA biosynthesis in AZ cells. The disruption of IAA transport increased the transcriptional activity of *LlZEP* in the first 4 h after treatment in both the distal and proximal areas of the AZ (Figure 4A). High mRNA content was also maintained 6 h after the TIBA application exclusively in the proximal region. No difference in *LlZEP* expression was noticed between the distal and proximal areas of the non-treated AZ (0 h). We observed an interesting pattern of ABA localization in the immunofluorescence experiments (Figure 4B,C). Six hours after the TIBA application, ABA was presented in small round structures (Figure 4C) situated directly in the AZ region, which formed a strip between the distal and proximal regions (Figure 4B).

**Figure 5:**
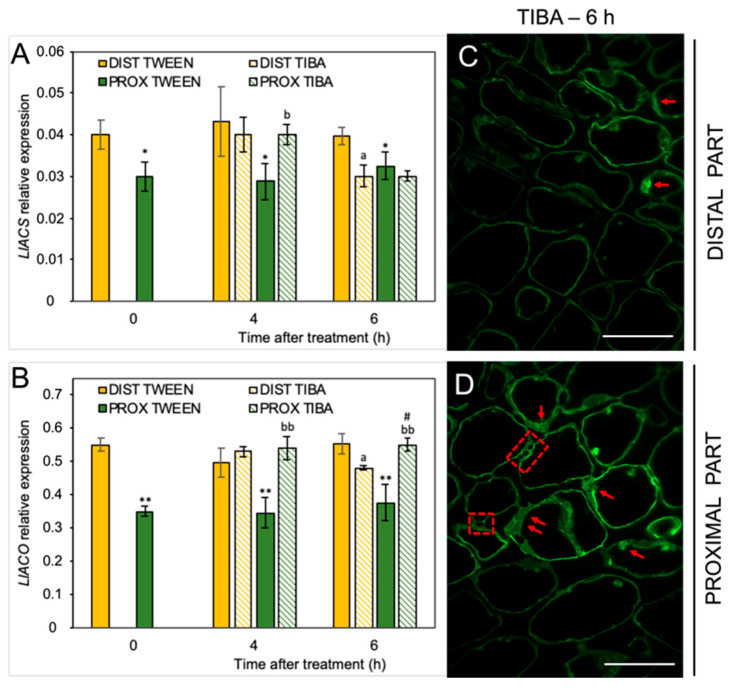
The disruption of polar auxin transport affected the ethylene (ET) biosynthesis pathway in different areas of the AZ. The relative level of 1-aminocyclopropane-1-carboxylic acid synthase (*LlACS*) transcripts (**A**) and 1-aminocyclopropane-1-carboxylic acid oxidase (*LlACO*) (**B**) genes were investigated using qPCR analysis. A solution of 2,3,5-triiodobenzoic acid (TIBA, 0.05 mM in 0.05% Tween 20) or 0.05% Tween 20 was applied directly on the inactive flower abscission zone (AZ). Then, the tissue fragments containing the AZ (≈1 mm below and ≈1 mm above) were dissected 4 h or 6 h after treatments and divided into a proximal (PROX) and a distal (DIST) part for further RNA isolation. *LlACT* (*ACTIN*) was used as an internal control for the normalization of gene expression. The average values and SD of three measurements per treatment are shown. *t*-test: ** *p* < 0.01 and * *p* < 0.05, proximal versus distal part in Tween 20-treated AZ for different times after application, ^a^ *p* < 0.05, TIBA-treated distal part versus Tween 20-treated distal part; ^bb^ *p* < 0.01 and ^b^ *p* < 0.05, TIBA-treated proximal part versus Tween 20-treated proximal part for different times after application, # *p* < 0.05, proximal versus distal part in the TIBA-treated AZ. The localization of the ET precursor 1-aminocyclopropane-1-carboxylic acid (ACC) in the distal (**C**) and proximal (**D**) areas of the flower AZ 6 h after the TIBA application. The green fluorescence signal indicates ACC localization (red arrowheads). Red squares indicate the presence of ACC in the possibly formed plasmodesmata. Scale bars: 40 µM.

Given the fact that IAA can alter ET biosynthesis in yellow lupine AZ [20] and considering the diverse pattern of IAA localization after the TIBA treatment, we decided to investigate the influence of PAT weakening on the components of the ET synthesis pathway. We have already proven that IAA stimulates *LlACS* expression and increases the level of the ET precursor 1-aminocyclopropane-1-carboxylic acid (ACC) in the flower AZ of yellow lupine [20]. In the present study, we showed that disturbance of the PAT via TIBA application led to *LlACS* down-regulation in the distal part (6 h) and up-regulation in the proximal region (4 h) (Figure 5A). The levels of both *LlACS* and *LlACO* transcripts were lower in the proximal area of the inactive, non-treated AZ (0 h) than in the distal one (Figure 5B). qPCR also confirmed that *LlACO* expression was enhanced after TIBA application in the proximal part but the treatment caused its down-regulation in the distal area of the AZ (Figure 5A,B). We then tested whether the localization of the ET precursor changed after inhibition of the auxin movement. The TIBA treatment caused a differential appearance of ACC in both areas of the AZ (Figure 5C,D). In the proximal part, ACC was localized in the thin layer of cytoplasm and in the potential plasmodesmata, which were formed between adjacent cells (Figure 5D). Such a strong signal was not observed in the distal region (Figure 5C). Collectively, a weakening of the PAT spatially affected the biosynthesis of strong phytohormonal inducers of flower abscission (ABA and ET).

### 2.5. Blocking of IAA Transport through the AZ Led to Fluctuations of the Redox Balance

**Figure 6:**
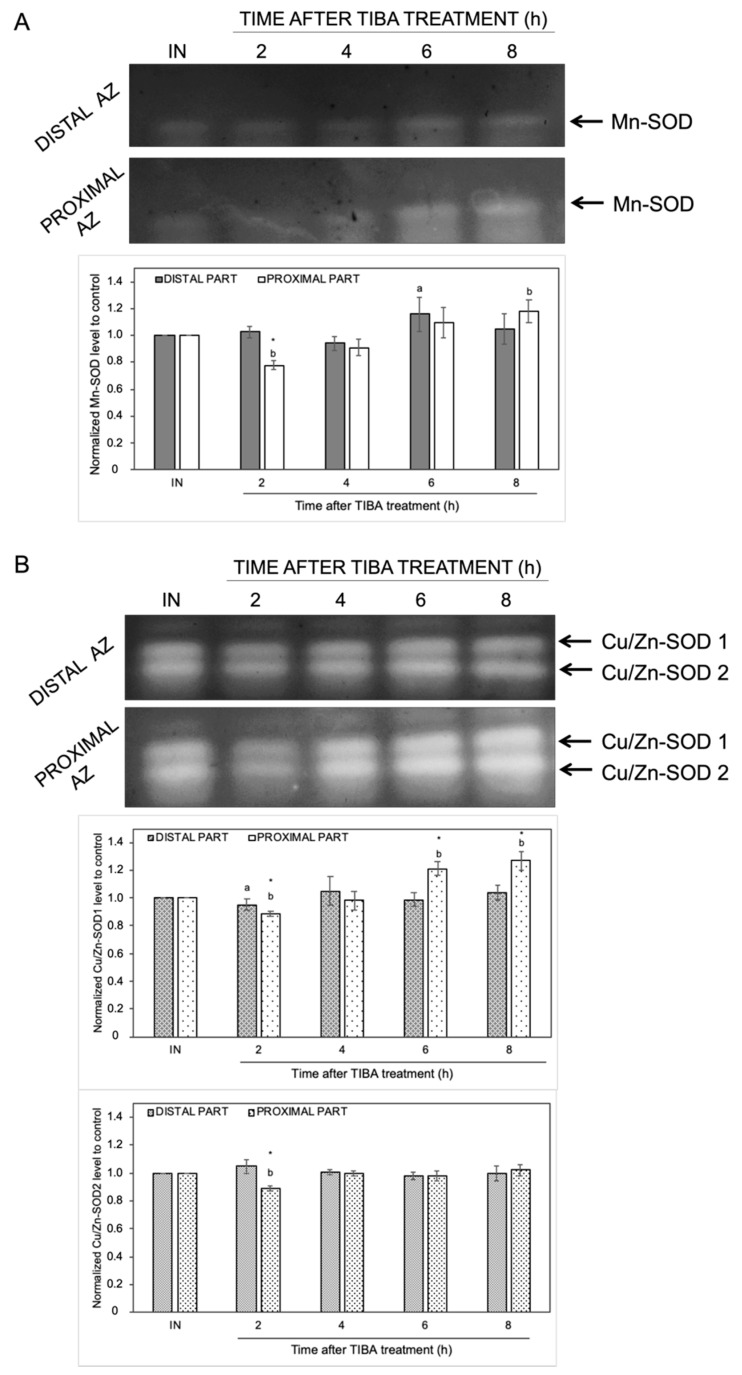
The response of distal and proximal parts of the flower AZ to the inhibition of polar auxin movement involved superoxide dismutase (SOD) activity. The SOD isoenzyme profile was obtained for the distal and proximal parts of the AZ. Tween 20 solution (0.05%) was applied directly to the inactive AZ (IN) and material was collected after 8 h. An inhibitor of polar auxin transport, namely 2,3,5-triiodobenzoic acid (TIBA, 0.05 mM in 0.05% Tween 20), was also applied to the IN AZ and tissues were excised 2, 4, 6, and 8 h after treatment. Mn-SOD (**A**) and two isoforms of Cu/Zn-SOD (**B**) were detected using in-gel assays (representative micrographs of nitroblue tetrazolium (NBT)-stained gel are presented). The charts below the pictures display the average densitometric data corresponding to the bands detected in three separate gels. Each band was quantified and expressed as the relative value of its intensity compared to the control AZ, which was considered to have a value of 1. Error bars indicate SEs. The values for each isoform were normalized to the control, namely the inactive AZ (IN). *t*-test: * *p* < 0.05, distal versus proximal part; ^a^ *p* < 0.05, treated distal part versus inactive distal part; ^b^ *p* < 0.05, treated proximal part versus inactive proximal part.

The changes in the endogenous levels of ABA and ET act as a signal of the appearance of stress conditions, and thus, ROS production could be evoked. We previously provided evidence that natural abscission of a lupine flower is linked to ROS accumulation in the AZ [19]. To better understand the effects of the inhibition of PAT on redox homeostasis, we focused on the examination of the activity of ROS scavenging enzymes and H_2_O_2_ production. In-gel assays of SOD activity revealed the presence of different isoforms: one identified as Mn-SOD and two as CuZn-SODs (Figure 6). The highest enzymatic activity of Mn-SOD was detected in the distal (6 h) and proximal (8 h) parts of the AZ cells (Figure 6A). When the AZ was exposed to TIBA, we observed an inhibition of Mn-SOD activity (2 h). The inhibition of PAT provoked various changes in Cu/Zn-SODs activity as well (Figure 6B). The activity of Cu/Zn-SOD1 and Cu/Zn-SOD2 isoforms was slightly inhibited in the second hour after TIBA application in the proximal area of AZ. The second isoform was not affected throughout the rest of the experiment and it was similar in both AZ parts. In turn, starting from 6 h after the TIBA treatment, the Cu/Zn-SOD1 activity gradually increased in the distal fragment (Figure 6B).

**Figure 7:**
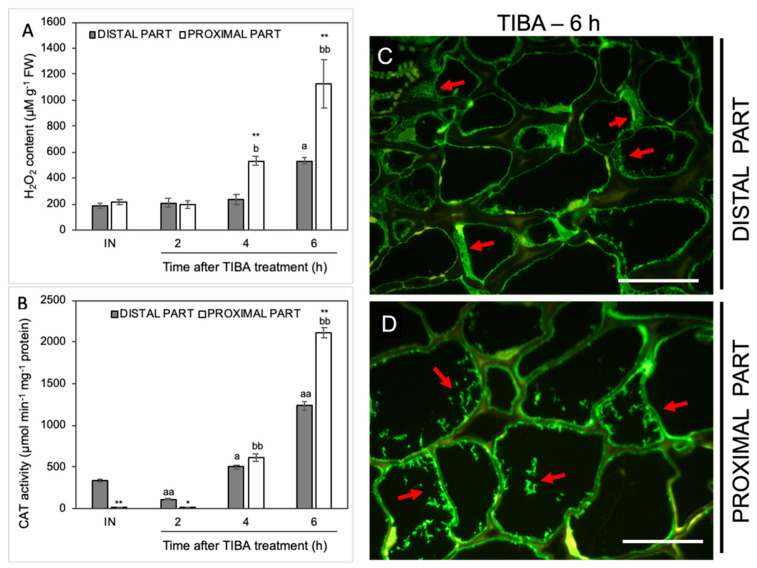
The level of H_2_O_2_ and the activity and localization of CAT were altered in various regions of the AZ under the influence of a polar auxin transport inhibitor. Tween 20 solution (0.05%) was applied directly to the inactive AZ (IN) and material was collected after 8 h. An inhibitor of polar auxin transport, namely 2,3,5-triiodobenzoic acid (TIBA, 0.05 mM in 0.05% Tween 20), was also applied to the IN AZ and tissues were excised 2, 4, 6, and 8 h after the treatment. The samples were used for the determination of H_2_O_2_ content (**A**) and catalase (CAT) activity (**B**). Values are presented as the mean of three biological replicates. Error bars indicate SEs. *t*-test: ** *p* < 0.01 and * *p* < 0.05, distal versus proximal part; ^aa^ *p* < 0.01 and ^a^ *p* < 0.05, treated distal part versus inactive distal part; ^bb^ *p* < 0.01 and ^b^ *p* < 0.05, treated proximal part versus inactive proximal part. The localization of CAT was performed using the specific antibody in the distal (**C**) and proximal (**D**) area of the flower AZ. Observations were carried out 6 h after the TIBA application. The green fluorescence signal marked by red arrows indicates the CAT presence. Scale bars: 40 µM.

In the next step, we analyzed the influence of TIBA on the level of H_2_O_2_ as a product of the reaction catalyzed by SOD. The H_2_O_2_ content was initially stable but started increasing from the fourth hour after the TIBA application in the proximal area of the AZ (Figure 7A). The maximum value was observed in the sixth hour. In this case, we also noticed a strong H_2_O_2_ accumulation in the distal part. Among all analyzed antioxidative enzymes, the most significant variations were observed for the activity of CAT, which is responsible for the enzymatic scavenging of H_2_O_2_ (Figure 7B). In the AZ before the TIBA treatment, the CAT activity was higher in the distal part than in the proximal one. Once the TIBA was locally applied, the CAT activity decreased in the distal area; however, it accelerated drastically afterward in both AZ regions. It should be noticed that the maximum value was observed in the proximal part of the AZ 6 h after the TIBA treatment. In this case, CAT was abundant in the whole AZ area but a different pattern of enzyme localization was observed (Figure 7C,D). Small fluorescence spots indicating CAT presence in the cells of the proximal part were observed (Figure 7D). In turn, the cytoplasm of cells of the distal side was filled with the enzyme (Figure 7C).

**Figure 8:**
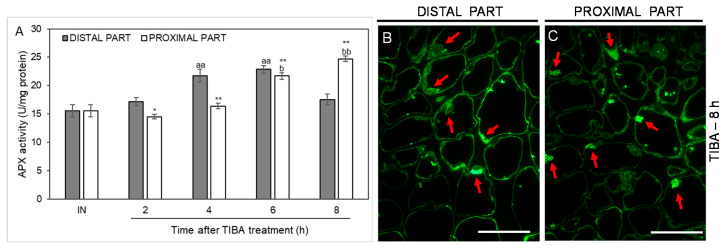
Time-course of ascorbate peroxidase (APX) activity and the enzyme localization in different regions of the AZ in response to the inhibition of polar auxin transport. Tween 20 solution (0.05%) was applied directly to the inactive AZ (IN) and material was collected after 8 h. An inhibitor of polar auxin transport, namely 2,3,5-triiodobenzoic acid (TIBA, 0.05 mM in 0.05% Tween 20), was also applied to the IN AZ and tissues were excised 2, 4, 6, and 8 h after treatment. APX activity was presented as the mean value of three biological replicates (**A**). Error bars indicate SEs. *t*-test: ** *p* < 0.01 and * *p* < 0.05, distal versus proximal part; ^aa^ *p* < 0.01, treated distal part versus inactive distal part; ^bb^ *p* < 0.01 and ^b^ *p* < 0.05, treated proximal part versus inactive proximal part. The localization of APX in the distal (**B**) and proximal (**C**) areas of the flower AZ. Observations were made 8 h after the TIBA application. The green fluorescence corresponds to APX presence (red arrowheads). Scale bars: 40 µM.

As an additional marker of antioxidant action in the AZ cells, we analyzed the activity of another ROS-scavenging enzyme, namely ascorbate peroxidase (APX), whose activity was up-regulated in the distal part of the AZ in the fourth and sixth hours after the TIBA application. In the case of the proximal part, the activity was shifted in time, with their maximum values measured at the last two time points considered (6 and 8 h after TIBA application). APX activity in the inactive areas of the AZ did not display noticeable changes between the proximal and distal parts (Figure 8). We also used a fluorescence-labeled antibody to detect APX localization. As Figure 8B presents, the enzyme was abundant in the peripheral region of cells forming the distal AZ. Observations of the proximal area revealed that APX accumulated in the vesicle-like structures or the cytoplasm (Figure 8C). Collectively, the blocking of PAT through the AZ was an abscission-triggering stimulus that strongly influenced redox homeostasis in specific areas of the flower AZ.

## 3. Discussion

Abscission is a genetics-dependent program but the crucial factor determining the time of organ separation is the changing level of phytohormones. During the last few decades, significant progress has been made toward understanding their mode of action following abscission. Initially, ABA was pointed to as a dominant factor responsible for organ detachment because of its role it stimulating flower abscission in *Gossypium hirsutum* [36]. Nowadays, ET, jasmonates, and gibberellins are considered to be stimulators of abscission, while brassinosteroids are considered to be a group of molecules responsible for inhibiting this process [37,38,39,40]. ET is generally accepted as a key effector of organ separation since it up-regulates enzymes required for cell wall remodeling. They are the main targets at the late stages of the abscission process related to a dissolution of a middle lamella between adjacent cells [25]. The stimulatory role of ET has been well documented in many species given that it is found in many different families, e.g., *Geraniaceae*, *Primulaceae*, *Ranunculaceae*, *Fabaceae*, and *Rosaceae* [22,23,41,42,43,44].

The activation of the AZ and the consequent organ separation requires dynamic hormonal interactions. Physiological and molecular analyses demonstrated that the time of abscission depends on the antagonistic action of ET and auxin [26]. The inhibition of auxin transport or flower excision accelerates pedicel separation, while the application of an inhibitor of the ET action reverses this effect. It has been proposed that the acquisition of sensitivity to ET by the AZ cells is linked to the modification of auxin-regulated genes [45]. For instance, fruit abscission in olive and melon is accompanied by down-regulation of genes encoding auxin receptors and transporters (transport inhibitor response 1 (*TIR*) and auxin efflux carriers (*AEC*)) [46,47]. The basipetal auxin movement prevents organ detachment, whereas its decay increases the sensitivity of the AZ to ET [1,48]. On the other hand, the abscission model proposed for mango fruits assumed that ET inhibits PAT, then the expression of ET receptor genes (*MiETR1* and *MiERS1*) is enhanced, and consequently, the sugar content in fruit decreases, and hence the abscission is stimulated [49]. It should also be emphasized that auxin can stimulate ET production, and in this way, causes organ separation [50,51,52]. We provided evidence that in *L. luteus*, IAA also affects ET biosynthesis genes (*LlACS*, *LlACO*) and causes an accumulation of the ACC, which is a precursor of ET in the flower AZ [20]. However, there are also data indicating the functioning of the ET-independent mechanism of auxin transport, e.g., during dark-induced leaf abscission in poplar [53]. 

As rapidly growing tissues, flowers are abundant sources of auxins, which are transported from the organ through the AZ. Such a distribution of auxins across the AZ area is important for the completion of abscission. When the auxin concentration at the distal side is lower than at the proximal one, abscission is favored, while the opposite pattern of this phytohormone distribution delays separation processes [31,32,45,54]. Our previous report also indicated that the activation of the flower AZ in yellow lupine is related to the spatiotemporal IAA maintenance of a gradient above and below the AZ [20]. This supports the significance of auxin distribution in the regulation of this process. However, the mechanism of the inhibition of auxin transport across the AZ following abscission remains to be elucidated. That is why we used TIBA in this study to stop auxin movement through AZ and we analyzed physiological, molecular, and biochemical changes evoked by this factor. 

As a weak aromatic acid, TIBA easily diffuses into cells, similar to IAA. TIBA and IAA bind to distinct but functionally related sites of the efflux transporter, and as a result, the movement of both molecules is inhibited [55,56]. TIBA has previously been shown to stimulate abscission in pea, orange, coleus, and rose [57,58,59,60]. Our physiological results showed that the direct application of TIBA onto the flower AZ in lupine resulted in excessive organ abscission (Figure 1A). The action of the inhibitor was reflected by curved stems, which became apparent 8 h and 24 h after treatment (Figure 1C,E). Furthermore, the AZ cells were anatomically distinct in response to TIBA (Figure 1H–J) and the visual symptoms were similar to those observed previously in the active AZ [29]. A heterogeneous cytoplasm and the presence of many vesicles may be related to the synthesis and transport of hydrolytic enzymes necessary for cell wall and membrane remodeling during abscission, e.g., cellulases, polygalacturonases, hydrolases, and expansins [47,61,62,63,64]. In bean, vesicles are enriched with callose that prevents the leakage of cytoplasm from broken tissues in the last steps of separation events [65]. Enlarged nuclei indicated the high metabolic activity of AZ cells (Figure 1J), similar to during leaf abscission in bean [66]. Collectively, TIBA application was sufficient to induce characteristic cellular changes that resulted in flower separation. The specificity of the inhibitor on auxin transport was also supported by GC-MS analysis of the auxin level (Figure 2C). Exogenous TIBA reversed the IAA localization pattern in the proximal and distal part of the AZ, which was confirmed by immunolocalization of the phytohormone (Figure 2D–G). The above experimental approach allowed us to further analyze the AZ-specific changes as those appearing in response to PAT inhibition. Although the history of studies on auxin participation in organ detachment is very long, according to our knowledge, no one has shown how inhibition of auxin redistribution across the AZ can affect the different metabolic pathways that are locally active in the AZ structure. We consider it essential to find out whether PAT inhibition might alter the subsequent molecular downstream elements that are expressed at the early abscission events. Once TIBA was applied, the up-regulation of *LlBOP*, *LlIDL*, *LlHSl*, and *LlMPK6* in the distal area of the AZ was observed, while most of them were expressed less efficiently in the proximal area when compared to the control (Figure 3). We also found that *LlIDL* expression was stimulated transiently, which is understandable since it encodes a ligand-protein that is a signal for the activation of the abscission-associated pathway. These findings are the first suggesting that the abscission pathway is activated in the upper part of the AZ and can be limited to the small fragment forming this structure. IDA-dependent abscission events have also been described in *Arabidopsis*, litchi, citrus, soybean, tomato, and bean [1,67,68,69,70,71]. On the one hand, data obtained in this study indicate that the induction of the molecular pathway of abscission in the distal side could lead to hormonal changes involving ABA and ET in the whole AZ area (Figure 4 and Figure 5), but on the other hand, suggest that these hormones-related events could be evoked simultaneously and independently. 

Meir et al. postulated that the IDA-HAE/HSL2 pathway can be ET-independent based on the analysis of ethylene-insensitive mutants in which organ abscission was delayed but not inhibited [72]. They proposed that IDA-HAE/HSL2 complex formation is crucial for the final stages of organ abscission, while ET is responsible for its initiation and progression. ET caused senescence in leaves and flowers in *Arabidopsis ida* mutants [68]; however, it cannot induce the abscission of floral organs, thus ET-independent functioning of IDA-HAE/HSL2 complex was suggested [67,68]. In turn, Tucker and Yang showed ET enhanced the expression of the *IDA* gene in non-AZ tissues, such as petioles and leaf blades in soybean [69]. Other recent studies have supported a positive influence of this phytohormone on *EgHSL* and *EgIDA* genes in the AZ of oil palm fruits [70], and *IDA-like* genes in the AZ cells of the fruitlet peduncle in litchi [71]. This kind of *LlIDL* regulation has also been demonstrated by us in the yellow lupine flower AZ [29]. Here, the increased expression of genes encoding molecular markers of flower separation was detected early, before the onset of abscission; thus, a direct effect of ET on IDL-dependent abscission pathway was highly possible.

Molecular changes in the lupine AZ were associated with spatial and temporal modulation of the homeostasis of ABA and ET (Figure 4 and Figure 5), which are the main stimulators of abscission [22,28]. The involvement of ABA in organ abscission has been well documented [4,73,74,75,76,77]. Its specific localization in AZ cells has also been reported [22]. Here, we present for the first time that ABA-mediated abscission depends on the PAT inhibition. The gene encoding the ABA-biosynthesis enzyme zeaxanthin epoxidase (*LlZEP*) was up-regulated in response to the TIBA treatment, especially in the proximal area of AZ (Figure 4A). We previously pointed out the involvement of *LlZEP* in the flower AZ activation processes and a positive correlation of its expression with ABA accumulation [21]. A similar relationship between *DcZEP1* expression and the ABA level in senescent carnation petals was observed [78]. Nevertheless, it cannot be excluded that stress hormones, like ABA and ET (produced in response to PAT inhibition), may also accelerate *LlZEP* expression. ET-dependent stimulation of *ZEP* was demonstrated in citrus fruits [79]. Interestingly, we observed in yellow lupine that ABA unexpectedly accumulated in the central area of the AZ only, and not in the proximal part of the AZ, as we had expected based on the *LlZEP* expression (Figure 4B,C). An explanation for the observed differences could be a direct positive influence of ABA on the ET biosynthesis elements involving *LlACS*, ACC, and *LlACO*, whose presence was strictly limited to the AZ cells, as we have previously shown [22]. Given these results, it was extremely interesting to investigate the effect of the inhibition of auxin movement through the AZ on the ET-biosynthesis pathway. To the best of our knowledge, there is no data regarding this issue in the available literature. The TIBA treatment led to down-regulation of *LlACS* and *LlACO* expression in the distal area of the AZ and up-regulation in the proximal part (Figure 5A,B), which was in line with the content of IAA (Figure 2B), and was already reported as a positive hormonal stimulator of both genes in lupine AZ [20]. The lower expression of *LlACS* (6 h) in the proximal part (Figure 5A) could be a result of a sufficient level of ACC present in this area, which was confirmed by immunofluorescence studies (Figure 5D). In this case, ACC could be synthesized by LlACS and/or released from cellular compartments. *LlACO* transcripts maintained a high level during this period (Figure 5B), which could be a consequence of substrate availability (Figure 5D) for an enzyme that *LlACS* encodes. Another explanation is the positive influence of the produced ET on the transcriptional activity of *LlACO*, which was experimentally confirmed in the lupine AZ (data not shown). The positive feedback loop in ET biosynthesis has been already reported in the literature [80,81,82,83]. There is also evidence showing that increasing *MdACS* and *MdACO* expression in active fruit AZ correlates with intensive ET production in apple [84], while high *ACO* activity, the accumulation of ACC, and ET production accompanied grape flower abscission [85]. During separation in peach and tomato, the increased expression of ACC oxidase genes was restricted to the fruit AZ cells, while in *Pelargonium*, it was up-regulated in the whole abscised sepals [86,87,88]. Summarizing this part, changes in the level of phytohormones along the AZ area in response to PAT inhibition is a signal for a plant that abscission processes are activated. 

It is well established that in many species, abscission is correlated with a burst of ROS in the AZ area [14,89,90,91]. We have also observed a strong accumulation of these compounds followed by the separation of lupine flowers [19]. Every plant cell has various enzymatic and non-enzymatic systems that regulate ROS levels. Among these, the enzymatic activity of SODs, CATs, and POXs seems to play a key role [15]. Fruit AZ transcriptome profiling in olive revealed that treatment with ethephon (an ethylene-releasing compound) induced the expression of *OeSOD1*, *OeCAT2*, and *OeCAT3* [91]. Girdling and defoliation leading to the separation of longan fruits up-regulated SOD, CAT, and POX activity in whole pedicels [89]. Given the different molecular and hormonal responses of distal and proximal cells of the AZ when the supply of auxin to the AZ is diminished, we aim to gain further insight into the redox balance in future research. The present study represents a first comprehensive analysis of the activity of the antioxidant systems induced by the inhibition of auxin movement in the AZ area. Overall, ROS-detoxification mechanisms involving SOD, CAT, and APX appears to be mainly activated in the proximal area of AZ (Figure 6, Figure 7 and Figure 8). SODs can be present in several isoforms characterized by the same catalytic specificity but with various kinetic properties and different migration rates on a gel [92]. In the flower AZ tissues of lupine, we detected the activity of three SODs isoforms that differ in their metal cofactors, namely one manganese (Mn-SOD) and two copper-zinc (Cu/Zn-SOD) isoforms (Figure 6). Among them, the lowest response to the TIBA treatment was observed for Cu/Zn-SOD2 (Figure 6B). In turn, the activity of Mn-SOD and Cu/Zn-SOD1 was quite similar in the 2, 4, and 8 h after the application of the PAT inhibitor (Figure 6A,B). Initially, in the proximal part, it was down-regulated, but then it increased in the final period considered, which implies the involvement of these isoforms in flower abscission evoked by the inhibition of auxin movement. SOD converts superoxide anion radicals generated by plasma membrane NADPH oxidase and/or during altered photosynthetic and mitochondrial electron transport into H_2_O_2_, which is further scavenged by CAT and POX. As expected, we observed a strong, gradual accumulation of H_2_O_2_ in the proximal area of the AZ (Figure 7A). The possible factor responsible for this action could be the IAA detected in this area (Figure 2C–G). The mode of auxin action in the AZ might be similar to that suggested for gravitropism responses, in which asymmetric movement of this hormone stimulates ROS generation [93]. A similar effect related to the up-regulation of auxin-dependent genes has been reported in response to oxidative stress conditions and the inhibition of auxin transport [94]. Thus, the changes that appeared in the lupine AZ after the TIBA treatment could be additionally accelerated by intensive H_2_O_2_ production. Its increasing content was consistent with the activity of CAT (Figure 7B) and APX (Figure 8A). The most crucial H_2_O_2_-detoxification mechanism in chloroplasts is the ascorbate–glutathione cycle, with APX as a key agent [95]. The thylakoid-bound form (tAPX) and the soluble, stroma-specific form (sAPX) of APX are localized in the chloroplast and catalyze the oxidation of ascorbate [96,97]. The second APX isoform scavenges the cytosolic H_2_O_2_ and exhibits higher specificity for phenols as an electron donor [97]. Together with the above-mentioned reports, we conclude that APX accumulated in the vesicle-like structures or the cytoplasm of the proximal AZ area (Figure 8C) could catalyze both ascorbate- and phenol-dependent oxidation. H_2_O_2_ has been shown to participate in the cross-linking reactions between lignin monomers and phenolic residues [98,99]. Recently, Lee et al. demonstrated that lignin acts as a molecular “brace” that supports the cell walls on the side of the separated organs [100]. Thus, there is a possibility that in the lupine AZ, active APX participates in the plant-cell-wall-loosening process and protective layers formation. Additionally, several lines of evidence point out that H_2_O_2_ may contribute to cellular signaling transduction pathways and modulate gene expression [101,102,103]. Given the lower activity of antioxidant enzymes compared to a stable level of H_2_O_2_ in the AZ (2 h), such a scenario cannot be excluded during the flower abscission evoked by the disruption of auxin transport (Figure 7 and Figure 8). In turn, the decreasing activity of SOD in the proximal area when compared to the distal one (2 h) can suggest that H_2_O_2_ synthesis may result from effects other than SOD-dependent reactions [104]. Taken together, the inhibition of IAA transport through AZ cells led to the mobilization of antioxidant pathways involving different enzymes that are active in a specific spatial and temporal manner.

**Figure 9:**
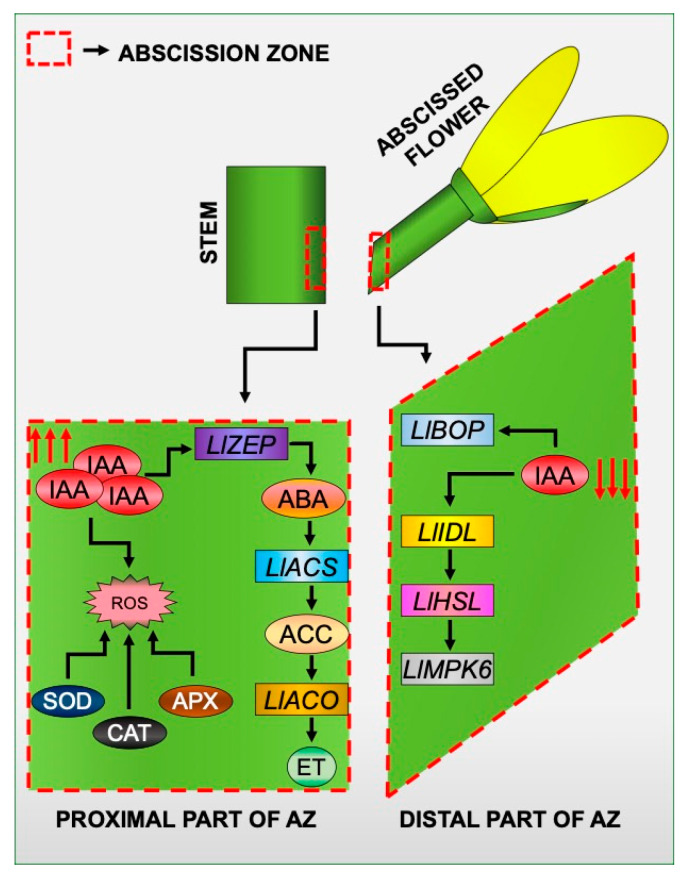
A proposed model for the specific action of molecular markers, phytohormones, and reactive oxygen species (ROS) in different areas of a flower AZ in response to the inhibition of polar auxin transport. Details are given in the text.

With all the results reported here and in our previous papers in mind, we created a model of the induction of tissue-specific changes in different areas of the flower AZ in response to the inhibition of the IAA movement (Figure 9). Disruption of IAA transport through the AZ cells led to the formation of an auxin gradient between different AZ areas. In these circumstances, the expression of elements of the molecular pathway that governs the time of abscission is specifically stimulated in the distal part of the AZ. It involves *LlBOP*, *LlIDL*, *LlHSL*, and LlMPK6 [21,28,29]. On the other hand, the response of proximal cells is associated with the appearance of ABA, and as a consequence, the acceleration of the biosynthesis rate of ET [22], which is the main abscission stimulator in lupine flowers [23]. IAA accumulated in the proximal area in response to the inhibition of PAT causes oxidative stress as a result of intensive H_2_O_2_ formation and activation of ROS scavenging mechanisms, such as SOD, CAT, and APX activity. We believe that our model of IAA-mediated asymmetric changes may have future applications in other plant abscission systems.

## 4. Materials and Methods 

### 4.1. Plant Material and Treatments 

The experiments in this study were carried out on yellow lupine (*Lupinus luteus* L.) Taper cultivar. Plants were grown in phytotrons under controlled light and temperature conditions, as previously described [28]. For the analyses, we used non-abscised tissue fragments containing inactive AZ (IN AZ). Our earlier studies revealed that this kind of AZ is present in the fully opened flowers (sixth stage of development) that have green pedicels. Precise morphological and anatomical criteria have already been described in our paper [28]. 

For assessing the effect of the inhibition of PAT on flower abscission the treatment with 2,3,5-triiodobenzoic acid (TIBA, Sigma-Aldrich, St. Louis, MO, USA, at a concentration of 0.05 mM in 0.05% Tween 20) was performed. The solution was applied precisely with a tiny brush on the inactive AZ fragments situated at the base of the flower pedicel. At the same time, each inactive AZ (IN) was treated with 0.05% Tween 20 only, which served as a control. Following subsequent hours after application of the substances, the flower pedicel and stem fragments containing the AZ were excised by hand under a binocular microscope using a razor blade (to a size of approx. 2 mm). Then, the harvested AZ was divided into two small pieces. The first, namely the distal region, was situated closer to the pedicel, and the second, namely the proximal region, was positioned below the AZ, near the stem (Supplementary Figure S1 and Figure 1A,B). The material was collected after different time intervals, which were given in the description of the result. The collected samples used for gene expression profiling (100 mg), measurement of hormone level (500 mg), or enzymatic assays (500 mg) were immediately frozen in liquid nitrogen and stored at −80 °C until the subsequent analysis steps. When making sections, fresh tissue fragments were fixed after the excision by following the methodology described in Section 4.4. The experiments were performed using three independent replicates (three pots × five plants). 

### 4.2. Physiological Experiment 

To assess the influence of the inhibition of PAT on flower shedding, a parallel set of lupines (15 plants in each biological replicate) were left for the TIBA treatment (0.05 mM in 0.05% Tween 20). The solution was applied directly onto the IN AZ. The control IN AZ was treated with 0.05% Tween 20. All plants were further monitored for the abortion rate. The number of detached flowers was counted 120 h after the application of the solutions. The experiment was performed using three biological replicates.

### 4.3. Expression Analysis Using Quantitative Real-Time PCR

For the RNA extraction, the distal and proximal AZ fragments were collected from the IN AZ (Tween-treated), as well as those treated with TIBA. Three biological replicates were harvested for each time point. The RNA was isolated and reverse transcribed while following a previously applied procedure [20]. After that, qPCR was performed with the LightCycler Real-Time PCR System using LightCyclerTaqMan Master (both provided by ROCHE Diagnostics GmbH, Mannheim, Germany). The primer pairs for amplification of the target and reference genes and sequences of hydrolysis probes are given in Supplementary Table S1. Conditions for optimized reactions were applied [23]. The relative expression values were calculated using LightCycler Real-Time PCR Systems software 4.1 (ROCHE Diagnostics GmbH, Mannheim, Germany). Obtained data were analyzed and presented with MS Office Excel (Microsoft, Redmond, WA, USA), and SigmaPlot 2001 v.7.0 (Systat Software Inc., San Jose, CA, USA). The qPCR reactions were performed in triplicate for each RNA template. 

### 4.4. Microscopy Sample Preparation

To perform the anatomical analysis, the AZ fragments were collected 24 h after treatment with TIBA or Tween 20. Next, they were fixed, dehydrated, embedded, sectioned, and placed on slides, as previously described [21]. The slides were used for histological analyses and immunolocalization studies.

### 4.5. Histological Analyses

Tissue sections of AZ were stained with Toluidine blue, following an earlier procedure [21]. The stained samples were examined with an Olympus BX 50 microscope (Olympus, Tokyo, Japan) equipped with an Olympus XC50 camera, using a 100× (numerical aperture: 1.4) immersion oil objective. 

### 4.6. Immunolocalization Assay

The sections on slides were subjected to immunofluorescence studies using antibodies provided by Agrisera (Vännäs, SWEDEN). In brief, sections were blocked in bovine serum albumin and incubated with primary antibodies. For anti-CAT (AS09 501), the protocol of Wilmowicz et al. was applied [21]. In the case of anti-IAA (AS06 193), we used our previous methodology [20], whereas reactions with anti-ACC (AS11 1800) and anti-ABA (AS09 446) antibodies were performed according to another study by our team [22]. Ascorbate peroxidase was detected using anti-APX (AS08 368) antibody diluted 1:50 in 1% bovine serum albumin (Sigma-Aldrich, St. Louis, MO, USA) in PBS buffer, pH 7.2 (chemicals for buffer preparation were provided by Sigma-Aldrich, St. Louis, MO, USA). For all primary antibodies, binding was detected with a goat anti-Rabbit (DyLight 488 conjugated) secondary antibody (AS09 633) that was diluted 1:250 in PBS buffer, pH 7.2 (incubation conditions: 2 h at 37 °C). Sections were examined with an Olympus BX 50 microscope equipped with an Olympus XC50 camera, using a 100× (numerical aperture: 1.4) immersion oil objective.

### 4.7. Determination of the Activity of Antioxidant Enzymatic System and H_2_O_2_ Measurements

Frozen AZ fragments (−80 °C) collected 0, 2, 4, 6, and 8 h after the TIBA treatment were subjected to enzyme assays. The control IN AZ was dissected and frozen 8 h after the Tween application. To perform the SOD activity measurements, the method based on nitroblue tetrazolium (NBT) reduction was applied. We obtained protein extracts and identified isoforms in PAGE, as described previously [105,106]. A comparison of the relative band intensity mm^−2^ to the one obtained for the Cu/Zn-SOD reference pattern of the defined activity of 4.4 U mg^−1^ protein (Sigma-Aldrich, St. Louis, MO, USA,) was applied to calculate the activity of a different SOD isoform band. In turn, the APX activity was evaluated in the extracts using the spectrophotometric method of Nakano and Asada [96], modified by Aksmann et al. [107]. In brief, it was based on the oxidation of pyrogallol measured by the increase in absorbance at 430 nm. The results were presented as U mg^−1^ protein (mmol pyrogallol oxidized min^−1^ mg^−1^ protein). Densitometric analysis was performed using Quantity1D (Bio-Rad, Hercules, California, USA) software. The CAT activity was determined spectrophotometrically according to Aksmann et al. [107] and the results were expressed as the amount of consumed substrate for CAT—H_2_O_2_ (μmol min^−1^ mg^−1^ protein). The reproducibility of all results was confirmed by making three independent replicates.

The concentration of H_2_O_2_ was analyzed, as described previously [21]. The results are presented as µmol H_2_O_2_ per gram of fresh weight. 

### 4.8. IAA Content Determination

The endogenous level of IAA in frozen (−80 °C) AZ fragments was examined using GC-MS according to a previously published methodology [20]. GC-MSSIM was performed by monitoring m/z 130 for IAA-methyl ester and m/z 132 for deuterium-labeled IAA-methyl ester (d_2_-IAA).

### 4.9. Statistical Analysis

The obtained data were subjected to statistical analysis performed using SigmaPlot 2001 v.5.0. The standard error (SE) and Student’s *t*-test were evaluated at significance levels of *p* < 0.05 and *p* < 0.01.

## Supplementary Materials

Supplementary materials can be found at https://www.mdpi.com/1422-0067/21/11/3815/s1, Figure S1: The methodology of treatment of the abscission zone (AZ) and further material collection. Treatment with 2,3,5-triiodobenzoic acid (TIBA, at concentration 0.05 mM in 0.05% Tween 20) was performed. The solution was applied precisely with a tiny brush on the AZ fragments situated at the base of the flower pedicel. At the same time, inactive AZ (IN) served as control and was treated with 0.05% Tween 20 only. Table S1: The primers and probes used in the qPCR reactions. All primers were synthesized by Genomed (Warsaw, Poland).

## Abbreviations

AZ	abscission zone
ABA	abscisic acid
ACS	aminocyclopropane-1-carboxylic acid synthase
ACO	aminocyclopropane-1-carboxylic acid oxidase
APX	ascorbate peroxidase
BOP	blade-on-petiole
CAT	catalase
ET	ethylene
HAE	HAESA
IAA	indole-3-acetic acid
IDA	inflorescence deficient in abscission
KNAT1	knotted-like from Arabidopsis thaliana 1
MPK6	mitogen-activated protein kinase 6
NBT	nitroblue tetrazolium
PAT	polar auxin transport
PCD	programmed cell death
POX	peroxidase
ROS	reactive oxygen species
SOD	superoxide dismutase
TIBA	2,3,5-triiodobenzoic acid
ZEP	zeaxanthin epoxidase

## References

[1] Estornell L.H., Agustí J., Merelo P., Talón M., Tadeo F.R. (2013). Elucidating mechanisms underlying organ abscission. Plant Sci..

[2] Sawicki M., Aït Barka E., Clément C., Vaillant-Gaveau N., Jacquard C. (2015). Cross-talk between environmental stresses and plant metabolism during reproductive organ abscission. J. Exp. Bot..

[3] Addicott F.T. (1982). Abscission.

[4] Aneja M., Gianfagna M., Ng E. (1999). The roles of abscisic acid and ethylene in the abscission and senescence of cocoa flowers. Plant Growth Regul..

[5] Dunlap J.R., Wang Y.T., Skaria A. (1994). Abscisic acid- and ethylene-induced defoliation of *Radermachera sinica* L.. Plant Growth Regul..

[6] Nakagami H., Pitzschke A., Hirt H. (2005). Emerging MAP kinase pathways in plant stress signalling. Trends Plant Sci..

[7] Cho S.K., Larue C.T., Chevalier D., Wang H., Jinn T.L., Zhang S., Walker J.C. (2008). Regulation of floral organ abscission in *Arabidopsis thaliana*. Proc. Natl. Acad. Sci. USA.

[8] Shi J.X., Malitsky S., De Oliveira S., Branigan C., Franke R.B., Schreiber L., Aharoni A. (2011). SHINE transcription factors act redundantly to pattern the archetypal surface of *Arabidopsis* flower organs. PLoS Genet..

[9] Shi C.L., Stenvik G.E., Vie A.K., Bones A.M., Pautot V., Proveniers M., Aalen R.B., Butenko M.A. (2011). Arabidopsis class I KNOTTED-like homeobox proteins act downstream in the IDA-HAE/HSL2 floral abscission signaling pathway. Plant Cell.

[10] Singh R., Singh S., Parihar P., Mishra R.K., Tripathi D.K., Singh V.P., Chauhan D.K., Prasad S.M. (2016). Reactive Oxygen Species (ROS): Beneficial Companions of Plants’ Developmental Processes. Front. Plant Sci..

[11] Michaeli R., Philosoph-Hadas S., Riov J., Meir S. (1999). Chilling-induced leaf abscission of Ixora coccinea plants. I. Induction by oxidative stress via increased sensitivity to ethylene. Physiol. Plant..

[12] Gapper C., Dolan L. (2006). Control of plant development by reactive oxygen species. Plant Physiol..

[13] Sakamoto M., Munemura I., Tomita R., Kobayashi K. (2008). Involvement of hydrogen peroxide in leaf abscission signaling, revealed by analysis with an in vitro abscission system in capsicum plants. Plant J..

[14] Sakamoto M., Munemura I., Tomita R., Kobayashi K. (2008). Reactive oxygen species in leaf abscission signaling. Plant Signal. Behav..

[15] Kärkönen A., Kuchitsu K. (2015). Reactive oxygen species in cell wall metabolism and development in plants. Phytochemistry.

[16] Alscher R.G., Erturk N., Heath L.S. (2002). Role of superoxide dismutases (SODs) in controlling oxidative stress in plants. J. Exp. Bot..

[17] Fernández-Pascual M., Pueyo J.J., de Felipe M.R., Golvano M.P., Lucas M.M. (2007). Singular features of the *Bradyrhizobium-Lupinus* symbiosis. Dyn. Soil Dyn. Plant.

[18] Coba de la Peña T., Pueyo J.J. (2012). Legumes in the reclamation of marginal soils, from cultivar and inoculant selection to transgenic approaches. Agron. Sustain. Dev..

[19] Kućko A., Smoliński D., Wilmowicz E., Florkiewicz A., de Dios Alché J. (2019). Spatio-temporal localization of *LlBOP* following early events of floral abscission in yellow lupine. Protoplasma.

[20] Kućko A., Wilmowicz E., Ostrowski M. (2019). Spatio-temporal IAA gradient is determined by interactions with ET and governs flower abscission. J. Plant Physiol..

[21] Wilmowicz E., Kućko A., Burchardt S., Przywieczerski T. (2019). Molecular and hormonal aspects of drought-triggered flower shedding in yellow lupine. Int. J. Mol. Sci..

[22] Wilmowicz E., Frankowski K., Kućko A., Świdziński M., de Alché J.D., Nowakowska A., Kopcewicz J. (2016). The influence of abscisic acid on the ethylene biosynthesis pathway in the functioning of the flower abscission zone in *Lupinus luteus*. J. Plant Physiol..

[23] Frankowski K., Kućko A., Zienkiewicz A., Zienkiewicz K., Kopcewicz J., de Dios J.D., Wilmowicz E. (2017). Ethylene-dependent effects on generative organ abscission of *Lupinus luteus*. Acta Soc. Bot. Pol..

[24] Bar-Dror T., Dermastia M., Kladnik A., Znidaric M.T., Novak M.P., Meir S., Burd S., Philosoph-Hadas S., Ori N., Sonego L. (2011). Programmed cell death occurs asymmetrically during abscission in tomato. Plant Cell.

[25] Merelo P., Agustí J., Arbona V., Costa M.L., Estornell L.H., Gómez-Cadenas A., Coimbra S., Gómez M.D., Pérez-Amador M.A., Domingo C. (2017). Cell wall remodeling in abscission zone cells during ethylene-promoted fruit abscission in Citrus. Front. Plant Sci..

[26] Taylor J.E., Whitelaw C.A. (2001). Signals in abscission. New Phytol..

[27] Meir S., Sundaresan S., Riov J., Agarwal I., Philosoph-Hadas S. (2015). Role of auxin depletion in abscission control. Stewart Postharvest Rev..

[28] Frankowski K., Kućko A., Zienkiewicz A., Zienkiewicz K., Alché J.D., Kopcewicz J., Wilmowicz E. (2015). Profiling the *BLADE-ON-PETIOLE* gene expression in the abscission zone of generative organs in *Lupinus luteus*. Acta Physiol Plant..

[29] Wilmowicz E., Kućko A., Ostrowski M. (2018). *INFLORESCENCE DEFICIENT IN ABSCISSION*-like is an abscission-associated and phytohormone-regulated gene in flower separation of *Lupinus luteus*. Plant Growth Regul..

[30] Eo J., Lee B.Y. (2011). Anatomical and histological changes in the fruit abscission zone of water dropwort (*Oenanthe stolonifera* DC.). Hortic. Environ. Biotechnol..

[31] Addicott F.T., Lynch R.S., Carns H.R. (1955). Auxin gradient theory of abscission regulation. Science.

[32] Louie D.S., Addicott F.T. (1970). Applied auxin gradients and abscission in explants. Plant Physiol..

[33] Goren R. (1993). Anatomical, physiological, and hormonal aspects of abscission in citrus. Hortic. Rev..

[34] Bonghi C., Tonutti P., Ramina A. (2000). Biochemical and molecular aspects of fruitlet abscission. Plant Growth Regul..

[35] Zhu H., Yuan R., Greene D.W., Beers E.P. (2010). Effects of 1-methylcyclopropene and naphthaleneacetic acid on fruit set and expression of genes related to ethylene biosynthesis and perception and cell wall degradation in apple. J. Am. Soc. Hortic. Sci..

[36] Ohkuma K., Lyon J.L., Addicott F.T., Smith O.E. (1963). Abscisin II, an abscission-accelerating substance from young cotton fruit. Science.

[37] Stutte G.W., Gage J. (1990). Gibberellin inhibits fruit abscission following seed abortion in peach. J. Am. Soc. Hort. Sci..

[38] Hartmond U., Yuan R., Burns J.K., Gran A., Kender W.J. (2000). Citrus fruit abscission induced by methyl-jasmonate. J. Am. Soc. Hort. Sci..

[39] Parra-Lobato M.C., Gomez-Jimenez M.C. (2011). Polyamine-induced modulation of genes involved in ethylene biosynthesis and signalling pathways and nitric oxide production during olive mature fruit abscission. J. Exp. Bot..

[40] Marciniak K., Kućko A., Wilmowicz E., Świdziński M., Przedniczek K., Kopcewicz J. (2018). Gibberellic acid affects the functioning of the flower abscission zone in *Lupinus luteus* via cooperation with the ethylene precursor independently of abscisic acid. J. Plant Physiol..

[41] Osborne D.J., Sargent J.A. (1976). The positional differentiation of abscission zones during the development of leaves of *Sambucus nigra* and the response of the cells to auxin and ethylene. Planta.

[42] Weis K.G., Goren R., Martin G.C., Webster B.D. (1988). Leaf and inflorescence abscission in olive. I. Regulation by ethylene and ethephon. Bot. Gaz..

[43] Woltering E.J., van Doorn W.G. (1988). Role of ethylene in senescence of petals - morphological and taxonomical relationships. J. Exp. Bot..

[44] Reid M.S., Wu M.J. (1992). Ethylene and flower senescence. Plant Growth Regul..

[45] Meir S., Philosoph-Hadas S., Sundaresan S., Selvaraj K.S.V., Burd S., Ophir R., Kochanek B., Reid M.S., Jiang C.Z., Lers A. (2010). Microarray analysis of the abscission-related transcriptome in the tomato flower abscission zone in response to auxin depletion. Plant Physiol..

[46] Gil-Amado J.A., Gomez-Jimenez M.C. (2013). Transcriptome analysis of mature fruit abscission control in olive. Plant Cell Physiol..

[47] Corbacho J., Romojaro F., Pech J.C., Latché A., Gomez-Jimenez M.C. (2013). Transcriptomic events involved in melon mature-fruit abscission comprise the sequential induction of cell-wall degrading genes coupled to a stimulation of endo and exocytosis. PLoS ONE.

[48] Sexton R., Roberts J.A. (1982). Cell biology of abscission. Annu. Rev. Plant Physiol..

[49] Hagemann M.H., Winterhagen P., Hegele M., Wünsche J.N. (2015). Ethephon induced abscission in mango: Physiological fruitlet responses. Front. Plant Sci..

[50] Abeles F.B., Rubinstein B. (1964). Regulation of ethylene evolution and leaf abscission by auxin. Plant Physiol..

[51] Morgan P.W., Hall W.C. (1964). Accelerated release of ethylene by cotton following application of indolyl-3-acetic acid. Nature.

[52] Jones M., Woodson W. (1999). Differential expression of three members of the 1- aminocyclopropane-1-carboxylate synthase gene family in carnation. Plant Physiol..

[53] Jin X., Zimmermann J., Polle A., Fischer U. (2015). Auxin is a long-range signal that acts independently of ethylene signaling on leaf abscission in *Populus*. Front. Plant Sci..

[54] Addicott F.T., Lynch R.S. (1951). Acceleration and retardation of abscission by indoleacetic acid. Science.

[55] Depta H., Rubery P.H. (1984). A comparative study of carrier participation in the transport of 2,3,5-triiodobenzoic acid, indole-3-acetic acid, and 2,4-dichlorophenoxyacetic acid by *Cucurbita pepo* L. hypocotyl segments. J. Plant Physiol..

[56] Teale W., Palme K. (2018). Naphthylphthalamic acid and the mechanism of polar auxin transport. J. Exp. Bot..

[57] Mao Z., Craker L.E., Decoteau D.R. (1989). Abscission in Coleus: Light and phytohormone control. J. Exp. Bot..

[58] Goszczynska D., Zieslin N. (1993). Abscission of flower peduncles in rose (Rosa × hybrida) plants and evolution of ethylene. J. Plant. Physiol..

[59] Yuan R., Hartmond U., Kender W.J. (2002). Naphthalene acetic acid and 2,3,5-triiodobenzoic acid affect the response of mature orange fruit to abscission chemicals. Hortic. Sci..

[60] Fišerová H., Šebánek J., Hradilík J., Procházka S. (2006). The effect of quercetine on leaf abscission of apple tree (*Malus domestica* Borkh.), growth of flax (*Linum usitatissimum* L.) and pea (*Pisum sativum* L.), and ethylene production. Plant Soil Environ..

[61] Taylor J.E., Tucker G.A., Lasslett Y., Smith C.J., Arnold C.M., Watson C.F., Schuch W., Grierson D., Roberts J.A. (1990). Polygalacturonase expression during leaf abscission of transgenic and normal tomato plants. Planta.

[62] Bonghi C., Rascio N., Ramina A., Casadoro G. (1992). Cellulase and polygalacturonase involvement in the abscission of leaf and fruit explants of peach. Plant Mol. Biol..

[63] Kalaitzis P., Solomos T., Tucker M.L. (1997). Three different polygalacturonases are expressed in tomato leaf and flower abscission, each with a different temporal expression pattern. Plant Physiol..

[64] Belfield E.J., Ruperti B., Roberts J.A., McQueen-Mason S. (2005). Changes in expansin activity and gene expression during ethylene-promoted leaflet abscission in *Sambucus nigra*. J. Exp. Bot..

[65] Sager R., Lee J.Y. (2014). Plasmodesmata in integrated cell signalling: Insights from development and environmental signals and stresses. J. Exp. Bot..

[66] McManus M.T., Thompson D.S., Merriman C., Lyne L., Osborne D.J. (1998). Transdifferentiation of mature cortical cells to functional abscission cells in *Phaseolus vulgaris* (L.). Plant Physiol..

[67] Jinn T.L., Stone J.M., Walker J.C. (2000). HAESA, an *Arabidopsis* leucine-rich repeat receptor kinase, controls floral organ abscission. Genes Dev..

[68] Butenko M.A., Patterson S.E., Grini P.E., Stenvik G.E., Amundsen S.S., Mandal A., Aalen R.B. (2003). INFLORESCENCE DEFICIENT IN ABSCISSION controls floral organ abscission in *Arabidopsis* and identifies a novel family of putative ligands in plants. Plant Cell.

[69] Tucker M.L., Yang R. (2012). IDA-like gene expression in soybean and tomato leaf abscission and requirement for a diffusible stelar abscission signal. AoB Plants.

[70] Stø I.M., Orr R.J., Fooyontphanich K., Jin X., Knutsen J.M., Fischer U., Tranbarger T.J., Nordal I., Aalen R.B. (2015). Conservation of the abscission signaling peptide IDA during Angiosperm evolution: Withstanding genome duplications and gain and loss of the receptors HAE/HSL2. Front. Plant Sci..

[71] Ying P., Li C., Liu X., Xia R., Zhao M., Li J. (2016). Identification and molecular characterization of an IDA-like gene from litchi, *LcIDL1*, whose ectopic expression promotes floral organ abscission in *Arabidopsis*. Sci. Rep..

[72] Meir S., Philosoph-Hadas S., Riov J., Tucker M.L., Patterson S.E., Roberts J.A. (2019). Re-evaluation of the ethylene-dependent and -independent pathways in the regulation of floral and organ abscission. J. Exp. Bot..

[73] Swanson B.T., Wilkins H.F., Weiser C.F., Klein I. (1975). Endogenous ethylene and abscisic acid relative to phytogerontology. Plant Physiol..

[74] Porter N.G. (1977). The role of abscisic acid in flower abscission of *Lupinus luteus*. Physiol. Plant..

[75] Sagee O., Erner Y. (1991). Gibberellins and abscisic acid content during flowering and fruit set of ‘Shamouti’ orange. Sci. Hortic..

[76] Vernieri P., Tagliasacchi A.M., Forino L., Lanfranchi A., Lorenzi R., Avanzi S. (1992). Abscisic acid levels and cell structure in single seed tissues of shedding affected fruits of *Malus domestica* Borkh.. J. Plant Physiol..

[77] Zacarias L., Talon M., Ben-Cheikh W., Lafuente M.T., Primo-Millo E. (1995). Abscisic acid increases in non-growing and paclobutrazol-treated fruits of seedless mandarins. Physiol. Plant..

[78] Nomura Y., Harada T., Morita S., Kubota S., Koshioka M., Yamaguchi H., Tanase K., Yagi M., Onozaki T., Satoh S. (2013). Role of ABA in triggering ethylene production in the gynoecium of senescing carnation flowers: Changes in ABA content and expression of genes for ABA biosynthesis and action. J. Japan. Soc. Hort. Sci..

[79] Rodrigo M.J., Zacarías L. (2007). Effect of postharvest ethylene treatment on carotenoid accumulation and the expression of carotenoid biosynthetic genes in the flavedo of orange (*Citrus sinensis* L. Osbeck) fruit. Postharvest Biol. Technol..

[80] Nakatsuka A., Murachi S., Okunishi H., Shiomi S., Nakano R., Kubo Y., Inaba A. (1998). Differential expression and internal feedback regulation of 1-aminocyclopropane-1-carboxylate synthase, 1-aminocyclopropane-1-carboxylate oxidase, and ethylene receptor genes in tomato fruit during development and ripening. Plant Physiol..

[81] Hiwasa K., Kinugasa Y., Amano S., Hashimoto A., Nakano R., Inaba A., Kubo Y. (2003). Ethylene is required for both the initiation and progression of softening in pear (*Pyrus communis* L.) fruit. J. Exp. Bot..

[82] Nakano R., Ogura E., Kubo Y., Inaba A. (2003). Ethylene biosynthesis in detached young persimmon fruit is initiated in calyx and modulated by water loss from the fruit. Plant Physiol..

[83] Inaba A., Liu X., Yokotani N., Yamane M., Lu W.J., Nakano R., Kubo Y. (2007). Differential feedback regulation of ethylene biosynthesis in pulp and peel tissues of banana fruit. J. Exp. Bot..

[84] Dal Cin V., Danesin M., Boschetti A., Dorigoni A., Ramina A. (2005). Ethylene biosynthesis and perception in apple fruitlet abscission (*Malus domestica* L. Borck). J. Exp. Bot..

[85] Hilt C., Bessis R. (2003). Abscission of grapevine fruitlets in relation to ethylene biosynthesis. Vitis.

[86] Clark D.G., Richards C., Hilloti Z., Lind-Iversen S., Brown K. (1997). Effect of pollination on accumulation of ACC synthase and ACC oxidase transcripts, ethylene production and flower petal abscission in geranium (*Pelargonium* x *hortorum* L.H. Bailey). Plant Mol. Biol..

[87] Ruperti B., Bonghi C., Rasori A., Ramina A., Tonutti P. (2001). Characterization and expression of two members of the peach 1-aminocyclopropane-1-carboxylate oxidase gene family. Physiol. Plant..

[88] Nakano T., Fujisawa M., Shima Y., Ito Y. (2013). Expression profiling of tomato pre- abscission pedicels provides insights into abscission zone properties including competence to respond to abscission signals. BMC Plant Biol..

[89] Yang Z., Zhong X., Fan Y., Wang H., Li J., Huang X. (2015). Burst of reactive oxygen species in pedicel-mediated fruit abscission after carbohydrate supply was cut off in longan (*Dimocarpus longan*). Front. Plant Sci..

[90] Roongsattham P., Morcillo F., Fooyontphanich K., Jantasuriyarat C., Tragoonrung S. (2016). Cellular and pectin dynamics during abscission zone development and ripe fruit abscission of the monocot oil palm. Front. Plant Sci..

[91] Goldental-Cohen S., Burstein C., Biton I., Ben Sasson S., Sadeh A., Many Y., Doron-Faigenboim A., Zemach H., Mugira Y., Schneider D. (2017). Ethephon induced oxidative stress in the olive leaf abscission zone enables development of a selective abscission compound. BMC Plant Biol..

[92] Kephart S.R. (1990). Starch gel electrophoresis of plant isoenzymes: A comparative analysis of techniques. Am. J. Bot..

[93] Joo J.H., Bae Y.S., Lee J.S. (2001). Role of auxin-induced reactive oxygen species in root gravitropism. Plant Physiol..

[94] Blomster T., Salojärvi J., Sipari N., Brosché M., Ahlfors R., Keinänen M., Overmyer K., Kangasjärvi J. (2011). Apoplastic reactive oxygen species transiently decrease auxin signaling and cause stress-induced morphogenic response in *Arabidopsis*. Plant Physiol..

[95] Asada K. (1992). Ascorbate peroxidase - a hydrogen peroxide-scavenging enzyme in plants. Physiol. Plant..

[96] Nakano Y., Asada K. (1981). Hydrogen peroxide is scavenged by ascorbate-specific peroxidase in spinach chloroplasts. Plant Cell Physiol..

[97] Chen G.X., Asada K. (1989). Ascorbate peroxidase in tea leaves: Occurrence of two isozymes and the differences in their enzymatic and molecular properties. Plant Cell Physiol..

[98] Brisson L.F., Tenhaken R., Lamb C. (1994). Function of oxidative cross-linking of cell wall structural proteins in plant disease resistance. Plant Cell.

[99] Schopfer P. (2001). Hydroxyl radical-induced cell-wall loosening in vitro and in vivo: Implications for the control of elongation growth. Plant J..

[100] Lee Y., Yoon T.H., Lee J., Jeon S.Y., Lee J.H., Lee M.K., Chen H., Yun J., Oh S.Y., Wen X. (2018). A lignin molecular brace controls precision processing of cell walls critical for surface integrity in *Arabidopsis*. Cell.

[101] García-Mata C., Lamattina L. (2013). Gasotransmitters are emerging as new guard cell signaling molecules and regulators of leaf gas exchange. Plant Sci..

[102] Shi C., Qi C., Ren H., Huang A., Hei S., She X. (2015). Ethylene mediates brassinosteroid-induced stomatal closure via Gα protein-activated hydrogen peroxide and nitric oxide production in *Arabidopsis*. Plant J..

[103] Li Z.G., Luo L.J., Sun Y.F. (2015). Signal crosstalk between nitric oxide and hydrogen sulfide may be involved in hydrogen peroxide –induced thermotolerance in maize seedlings. Russ. J. Plant Physiol..

[104] Niu L., Liao W. (2016). Hydrogen peroxide signaling in plant development and abiotic responses: Crosstalk with nitric oxide and calcium. Front. Plant Sci..

[105] Pokora W., Reszka J., Tukaj Z. (2003). Activities of super-oxide dismutase (SOD) isoforms during growth of *Scenedesmus* (Chlorophyta) species and strains grown in bath-cultures. Acta Physiol. Plant..

[106] Tukaj Z., Pokora W. (2006). Individual and combined effect of anthracene, cadmium and chloridazon on growth and activity of SOD isoforms in three *Scenedesmus* species. Ecotoxicol. Environ. Saf..

[107] Aksmann A., Pokora W., Baścik-Remisiewicz A., Dettlaff-Pokora A., Tukaj Z. (2016). High hydrogen peroxide production and antioxidative enzymes expression in the *Chlamydomonas reinhardtii cia3* mutant with an increased tolerance to cadmium and anthracene. Phycol. Res..

