# Supplementary material for: Disruption of the Auxin Gradient in the Abscission Zone Area Evokes Asymmetrical Changes Leading to Flower Separation in Yellow Lupine
Source: Int J Mol Sci. 2020 May 27;21(11):3815. doi: 10.3390/ijms21113815 (PMC7312349; doi:10.3390/ijms21113815)
Supplement: Supplementary file 1 [file ijms-21-03815-s001.pdf]

Figure S1. The methodology of treatment of abscission zone (AZ) and further material collection. Treatment with 2,3,5-triiodobenzoic acid (TIBA, at concentration 0.05 mM in 0.05% Tween 20) was performed. The solution was applied precisely with a tiny brush on the AZ fragments situated at the base of the flower pedicel. At the same time, inactive AZ (IN) served as control was treated with 0.05% Tween 20 only.

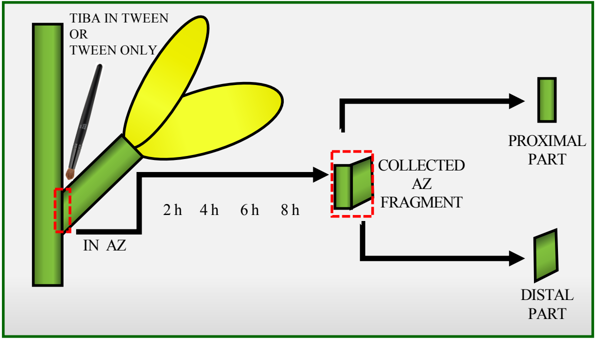

Table S1. The primers and probes used in the qPCR reactions.
All primers were synthesized by Genomed (Warsaw, Poland).

**Gene**	**GeneBank accession no.**	**Primer sequence 5'-3'**	**UPL probe no.**
LlACT	KP257588	F: TAATGGTTGGGATGGGTCAG<br>R: CAAGGTGAGAATACCCCTCT	165
LlACS	KF573522	F: TTCATTCAAGAAGGCAATGGT<br>R: GGTTTGGGTCAAAAGTCACC	53
LlACO	KF573523	F: GTGATGAAGGAATTTGCACAAG<br>R: CCAAGGTTTTCACACAGCAA	53
LlBOP	KC792647	F: GGAACTCGTCAAGCTCATGG<br>R: GGCGTAGTGTAAGGCCAATG	133
LlIDL	KT716180	F: ACAGCACCATGGTCACTTTTC<br>R: TTGTGTTTCCTTGAGGGAGTG	137
LlZEP	KF887951	F: GGGTTTCCGGTTCTTGGTAT<br>R: AAAGCCATTCGACTAATAACTCTTG	165
